# Tumor Treating Fields (TTFields) Reversibly Permeabilize the Blood–Brain Barrier In Vitro and In Vivo

**DOI:** 10.3390/biom12101348

**Published:** 2022-09-22

**Authors:** Ellaine Salvador, Almuth F. Kessler, Dominik Domröse, Julia Hörmann, Clara Schaeffer, Aiste Giniunaite, Malgorzata Burek, Catherine Tempel-Brami, Tali Voloshin, Alexandra Volodin, Adel Zeidan, Moshe Giladi, Ralf-Ingo Ernestus, Mario Löhr, Carola Y. Förster, Carsten Hagemann

**Affiliations:** 1 Department of Neurosurgery, Section Experimental Neurosurgery, University of Würzburg, D-97080 Würzburg, Germany; 2 Department of Anaesthesiology, Intensive Care, Emergency and Pain Medicine, University of Würzburg, D-97080 Würzburg, Germany; 3 Novocure Ltd., Haifa 3190500, Israel

**Keywords:** blood–brain barrier, TTFields, CNS disorders

## Abstract

Despite the availability of numerous therapeutic substances that could potentially target CNS disorders, an inability of these agents to cross the restrictive blood–brain barrier (BBB) limits their clinical utility. Novel strategies to overcome the BBB are therefore needed to improve drug delivery. We report, for the first time, how Tumor Treating Fields (TTFields), approved for glioblastoma (GBM), affect the BBB’s integrity and permeability. Here, we treated murine microvascular cerebellar endothelial cells (cerebEND) with 100–300 kHz TTFields for up to 72 h and analyzed the expression of barrier proteins by immunofluorescence staining and Western blot. In vivo, compounds normally unable to cross the BBB were traced in healthy rat brain following TTFields administration at 100 kHz. The effects were analyzed via MRI and immunohistochemical staining of tight-junction proteins. Furthermore, GBM tumor-bearing rats were treated with paclitaxel (PTX), a chemotherapeutic normally restricted by the BBB combined with TTFields at 100 kHz. The tumor volume was reduced with TTFields plus PTX, relative to either treatment alone. In vitro, we demonstrate that TTFields transiently disrupted BBB function at 100 kHz through a Rho kinase-mediated tight junction claudin-5 phosphorylation pathway. Altogether, if translated into clinical use, TTFields could represent a novel CNS drug delivery strategy.

## 1. Introduction

The homeostasis of the brain microenvironment is maintained and protected by the blood–brain barrier (BBB). The functions of the BBB are jointly orchestrated by endothelial cells forming the capillaries of the brain, astrocyte end-feet, pericytes, adjacent neurons, and a noncellular basement membrane. Molecular transport across the barrier is tightly restricted and regulated [1,2,3]. In this regard, one of the greatest hurdles faced by modern medicine in delivering drugs to the CNS is the BBB. Molecule drugs larger than 400 Da, such as the potent anticancer agents paclitaxel (PTX), methotrexate, and vincristine, are unable to reach the brain [4,5,6].

Adherens, gap, and tight junctions (TJs) seal the paracellular gaps between neighboring endothelial cells of the BBB. Adherens junctions lie closest to the basolateral membrane and are connected to the cytoskeleton via plaque proteins. They modulate receptor signaling and regulate the transendothelial migration of leukocytes. Gap junctions form connexons between endothelial cells and are known to maintain tight junction integrity. Located closest to the apical membrane, TJs limit the paracellular passage of molecules across the BBB, forming the so-called zonula occludens [7,8,9,10]. TJs are composed of integral membrane proteins, namely, claudins, occludin, and junctional adhesion molecules as well as cytoplasmic accessory proteins including Zonula occludens-1, -2, and -3 (ZO-1, ZO-2, and ZO-3) and cingulin [11,12,13]. Claudin-5 is a major TJ protein that regulates macromolecular transport through the BBB by forming elliptic meshes that restrict the passage of macromolecules [14]. Occludin, another TJ protein, is also suggested to play a role in TJ stability and barrier function [15]. ZO proteins (e.g., ZO-1) are essential for TJs, as they link the tight junctional molecules claudin and occludin to intracellular actin and the cytoskeleton via cingulin [16,17]. Together with TJs, the BBB is sealed by intercellular junctions such as the platelet endothelial cell adhesion molecule-1 (PECAM-1). Concentrated at cell–cell borders, PECAM-1 regulates vascular permeability and functions as an endothelial mechanosensor.

Due to the restrictions imposed by the BBB in allowing macromolecular drugs to reach the brain, advanced drug delivery methods are being widely investigated to enhance transport across the BBB including focused ultrasound, osmotic disruption, drug delivery vehicles, and pulsed electric fields (PEFs) [18,19,20]. However, these modalities have not been widely adopted in the clinic.

Tumor Treating Fields (TTFields) are low-intensity (1–3 V/cm), intermediate-frequency (100–300 kHz), alternating electric fields delivered loco-regionally via arrays placed on the skin, with known antimitotic effects on cancer cells. TTFields are frequency tuned per indication (e.g., 150 kHz for breast, pancreatic, and non-small cell lung cancer [21,22,23]), and they are approved as therapy for glioblastoma (GBM) patients when applied at 200 kHz [23,24,25,26,27]. The addition of TTFields (200 kHz) to maintenance temozolomide (TMZ) chemotherapy resulted in statistically significant improvements in progression-free and overall survival relative to TMZ alone in a randomized Phase III clinical trial [28]. The most commonly reported TTFields-related adverse events are mild to moderate array-associated skin reactions, which are manageable in the majority of cases. Other less prevalent TTFields-related adverse events in patients with GBM include under-array heat sensation, electric sensation, and headache [28,29].

While TMZ proves to be an efficient drug against GBM, other potent drugs that could potentially target GBM are not able to convey their efficacy due to the selectivity of the BBB. As such, effective strategies for delivering these drugs to the brain without being restricted by the BBB or introducing major adverse events are necessary.

We hypothesized that TTFields may have the potential to facilitate drugs crossing the BBB. A recent study demonstrated that TTFields application leads to the reorganization of the microtubule network resulting in initiation of the guanine nucleotide exchange factor (GEF)-H1/Rho/Rho kinase (ROCK) signaling pathway in cancer cells [30]. Activation of this pathway may also affect the function of the BBB. Experimental data show that claudin-5 silencing enhanced BBB permeability in the human brain [31]. Several groups have independently shown that claudin-5 can be modified by ROCK signaling-induced phosphorylation at threonine 207 (T207) [32,33,34,35]. It has been demonstrated that this phosphorylation may interfere with claudin-5 association with other TJ-anchoring molecules (e.g., ZO-1), leading to claudin-5 destabilization at the plasma membrane, reducing barrier tightness and potentially allowing for the passage of therapeutics. Considering the favorable safety profile of TTFields, this novel modality could address the need for new strategies to improve drug delivery to the brain. Hence, we herein assessed the effects of TTFields on BBB integrity and permeability both in vitro and in vivo to determine their potential as a means to disrupt the BBB for enhanced drug delivery.

## 2. Materials and Methods

### 2.1. Assessment of TTFields’ Effects In Vitro

The full methods are available in the Supplementary Materials. Briefly, the effects of TTFields were assessed in vitro using immortalized murine cerebellar microvascular endothelial cells (cerebEND) to which TTFields were applied for 24–72 h at various frequencies and intensities using the inovitro™ TTFields Lab Bench System (Novocure^®^). Following exposure to TTFields, cells were allowed to recover for 24–96 h before being subjected to further assays.

Immunofluorescence was used to assess the integrity of the cell monolayer after exposure to TTFields. Specifically, claudin-5, ZO-1, and PECAM-1 were stained and examined using microscopy. Furthermore, cell fractionation assays were used to evaluate the extent (if any) of tight junction protein delocalization, following exposure to TTFields.

The mechanism by which TTFields disrupts the integrity of the BBB was investigated using Western blots and commercially available activation assays specific for the GEF-H1/Rho/ROCK pathway.

Lastly, a TdT-mediated dUTP-biotin nick end labeling (TUNEL) assay was used to determine whether TTFields induced apoptosis in the noncancerous cerebEND cells.

Changes in the permeability of the cell monolayer were characterized using transendothelial electrical resistance (TEER) and a 4 kDa FITC-dextran assay to visualize movement of fluorescent dye through the monolayer. Allocation of samples to treatment and control groups was conducted by the researchers by random assignment prior to the start of the experiment (i.e., for in vitro experiments, when the cells were seeded).

### 2.2. Assessment of TTFields Effects In Vivo

The full methods are available in the Supplementary Materials. Briefly, female Sprague–Dawley and Fischer rats, the latter bearing intracranial GBM, were treated with TTFields at 100 kHz as previously described [36]. Changes in the permeability of the BBB were assessed using microscopic evaluation of brain cryosections and MRI scans conducted before, during, and after TTFields treatment.

### 2.3. Statistical Analysis

All in vitro experiments were conducted at least three times. No data were excluded from the statistical analyses. In vitro and in vivo data were analyzed through the GraphPad Prism 6 and 8 software (GraphPad Software, Inc., San Diego, CA, USA), respectively. Statistical significance was defined by unpaired two-tailed Student’s *t*-tests and analysis of variance (ANOVA), where applicable. *p* < 0.05 was considered significant. For nonparametric data, significance was tested with a Mann–Whitney test with a 95% confidence interval.

## 3. Results

### 3.1. TTFields Effects on the BBB Were Dependent on the Frequency, Intensity, and Duration of the Treatment

**Figure 1:**
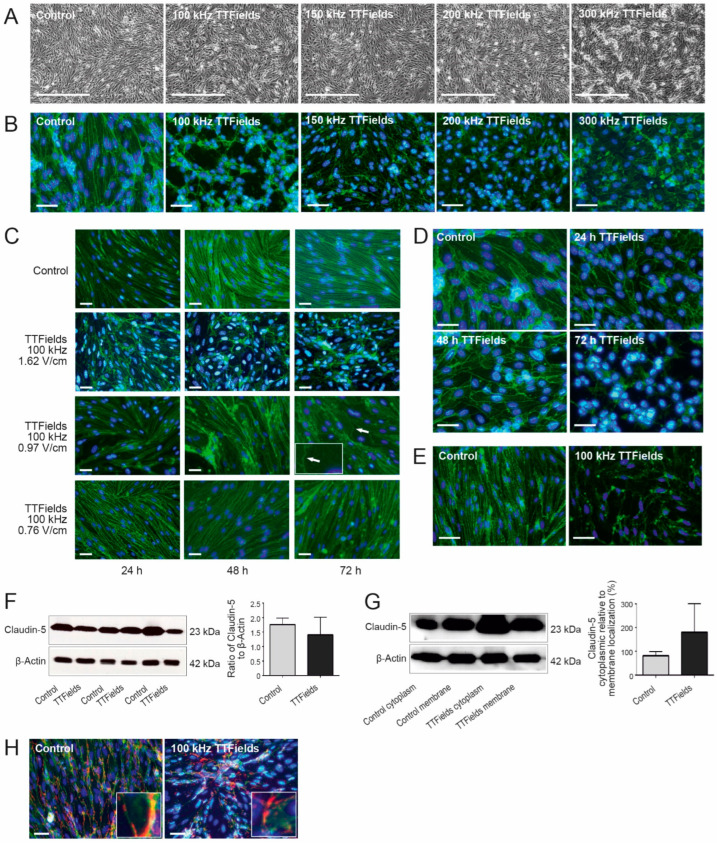
TTFields modified cerebEND structures by delocalizing claudin-5 and ZO-1 but not PECAM-1, with the strongest effects after at least 72 h: (**A**) light microscopy images of confluent monolayers of cerebEND cells untreated or treated with TTFields at 100–300 kHz for 72 h. Scale bar = 500 µm. (**B**) Immunofluorescence staining of cells under the same treatment conditions with antibody against claudin-5 (green). (**C**) cerebEND cells treated with 100 kHz TTFields for 24–72 h at TTFields intensities of 1.62, 0.97, and 0.76 V/cm, respectively. The arrow points to a large cell with frayed outlines in the membrane, which is further highlighted by the inlay. (**D**) Claudin-5 staining in a time course of cerebEND cells treated with TTFields at 100 kHz for 24, 48, and 72 h. (**E**) Immunofluorescence staining of ZO-1 (green) in cerebEND cells treated with TTFields at 100 kHz for 72 h. (**F**,**G**) Western blot and densitometric analyses of TJ protein claudin-5 expression in cerebEND cells treated with TTFields at 100 kHz for 72 h. While (**F**) shows the total cell lysates, (**G**) visualizes the difference of cytoplasmic- versus membrane-localized claudin-5. Values shown are the mean of three independent experiments normalized to β-actin. (**H**) Claudin-5 (green) and PECAM-1 (red) double staining of cerebEND cells. The magnified inlays clarify colocalization of claudin-5 and PECAM-1 in the control but not the cells to which TTFields were administered. In (**B**–**E**,**H**), nuclear staining was performed with DAPI (blue), magnification 40×, scale bar = 200 µm. Shown are representative images of *n* = 3 independent experiments. The claudin-5 antibody used in (**B**–**D**,**H**) was conjugated to Alexa Fluor 488 (green).

In order to initially define the effects of TTFields on the BBB, we conducted light microscopic analysis of murine cerebellum-derived microvascular endothelial cells (cerebEND) following TTFields administration at various frequencies. Under control conditions, cells grew parallel to each other in a confluent monolayer and appeared elongated and narrow. However, when cells were subjected to treatment with TTFields, the linear growth pattern was replaced by the appearance of swirls (Figure 1A). To better facilitate visualization, the tight junction proteins, claudin-5 and ZO-1, which are typically located at the boundaries between endothelial cells, were stained. Immunofluorescence imaging confirmed that following treatment with TTFields, the cells lost their elongated form and took on variable cell shapes (Figure 1B). All TTFields frequencies (100–300 kHz) applied to the cells resulted in the disruption and alteration of cellular morphology. However, the most dramatic visually observable effects were detected with TTFields at 100 kHz. Owing to this, succeeding experiments were conducted using TTFields at a frequency of 100 kHz.

In addition to being frequency specific, the cytotoxic effect of TTFields has been shown to be intensity dependent [23,27]. In order to test whether this was also the case for BBB opening, we evaluated TTFields at intensities of 1.62, 0.97, and 0.76 V/cm root mean square (RMS). Claudin-5 immunofluorescence staining of cerebEND cells treated with TTFields at an intensity of 0.76 V/cm RMS appeared similar to the control, with long, narrow cells that were observed to be tapered at the ends, independent of the duration of TTFields administration (24–72 h). Nevertheless, the presence of frayed outlines in the membranes was notable. CerebEND cells that were treated with TTFields at an intensity of 0.97 V/cm RMS revealed some morphological deformation regardless of the duration of TTFields application. Cells lost their fusiform appearance and were transformed to larger cells with frayed boundaries. The effects on cellular morphology were most dramatic following application of TTFields at an intensity of 1.62 V/cm RMS, where the distribution of claudin-5 appeared most disturbed. Therefore, there was clear intensity dependence in the cells’ response (Figure 1C).

As a next step, we investigated the time point by which TTFields at 100 kHz and 1.62 V/cm was most effective. CerebEND cells were treated with TTFields for 24, 48, and 72 h. After 24 h, cells already appeared disrupted as evidenced by immunofluorescence staining of the TJ protein claudin-5. The effects generated after 48 h were comparable to those after 24 h. However, the most striking effects were observed after 72 h (Figure 1D). Based on these results, further experiments were conducted using TTFields at 100 kHz for 72 h.

### 3.2. TTFields Altered BBB Morphology by Delocalizing Claudin-5 and ZO-1 but Not PECAM-1

Immunofluorescence staining of cerebEND cells following TTFields application revealed that the cell boundaries were disrupted, and claudin-5 as well as ZO-1 were delocalized from within the membrane to the cytoplasm (Figure 1B,E). To assess whether the alteration in cell morphology was influenced by the expression level of claudin-5, which is the major TJ protein expressed by cerebEND cells [37], a Western blot analysis was performed. The results revealed that there was no significant difference in claudin-5 protein expression between the control and cells treated with TTFields (Figure 1F). When separating the membrane from the cytoplasmic fraction of the cells, there appeared to be more claudin-5 in the cytoplasm than in the membrane of cells treated with TTFields (% of claudin-5 in cytoplasmic fraction relative to the membrane fraction). Although not statistically significant (*p* = 0.1644), there was an apparent delocalization of claudin-5 from the cell boundaries to the cytoplasm (Figure 1G).

We also analyzed changes in the localization of PECAM-1, which regulates vascular integrity. In the control samples, dual staining of claudin-5 and PECAM-1 resulted in staining overlap, implying colocalization of the two proteins at the cell boundaries as reported previously [38]. In contrast, in cells treated with TTFields, claudin-5 was delocalized away from the points of colocalization, while PECAM-1 remained along the cell boundaries (Figure 1H).

### 3.3. TTFields Application Promoted Phosphorylation of Claudin-5 in a Rho Kinase (ROCK)-Dependent Manner

**Figure 2:**
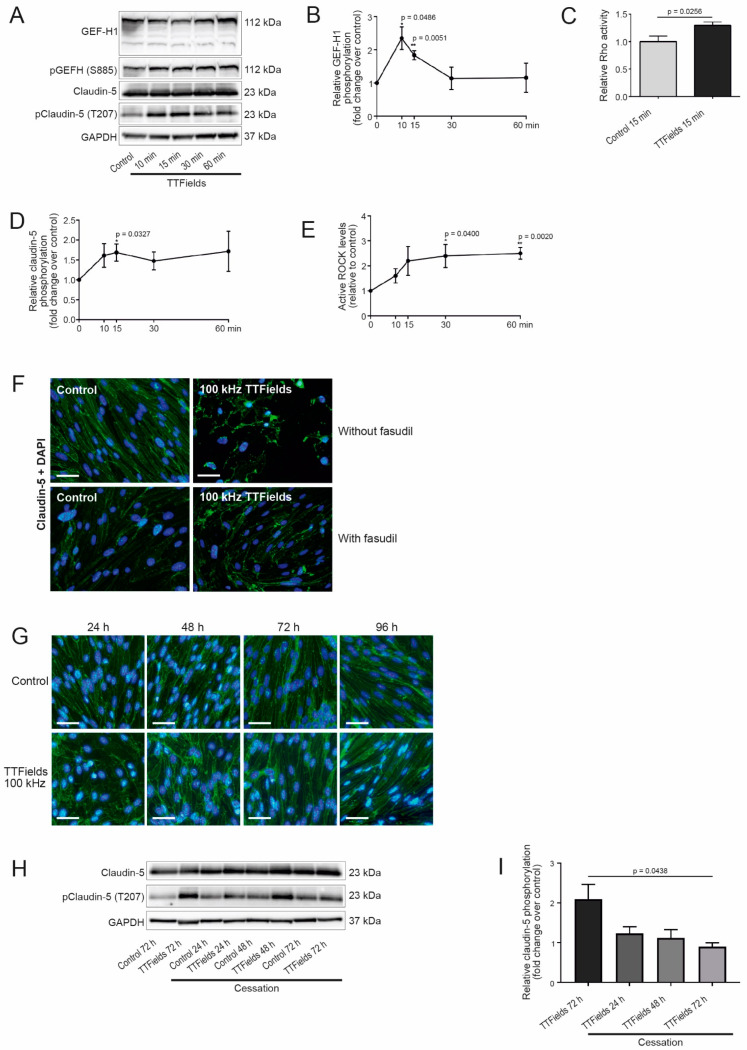
TTFields application promoted phosphorylation of claudin-5 in a ROCK-dependent manner. (**A**) Western blot analysis of GEF-H1 phosphorylated on S885 and claudin-5 phosphorylated on T207 in control and TTFields-treated cells. (**B**) Quantification of normalized relative levels of phosphorylated compared to total levels of GEF-H1 from three to four Western blots for each condition. Asterisks indicate significant differences compared to the control. (**C**) RhoA activation was measured by the RhoA G-LISA activation assay. (**D**) Quantification of normalized relative levels of phosphorylated compared to total levels of claudin-5 from three to four Western blots for each condition. Asterisks indicate significant differences compared to the control. (**E**) ROCK activity expressed as the ratio of the OD of treated cells to the OD of untreated cells. Asterisks indicate significant differences compared to the control. (**F**) Inhibition of ROCK with 10 µM fasudil in cerebEND cells treated with TTFields at 100 kHz for 72 h and untreated controls. Immunofluorescence staining with antibody against claudin-5 (green). Nuclear staining with DAPI (blue), scale bar = 50 µm. Shown are representative images of *n* = 3 independent experiments. (**G**) Cells treated with TTFields at 100 kHz for 72 h were allowed to recover for 24, 48, 72, and 96 h at 37 °C. They were then stained with antibody against claudin-5 conjugated to Alexa Fluor 488 (green). Nuclei were stained with DAPI (blue), magnification 40×, scale bar = 200 µm. Shown are representative images of *n* = 3 independent experiments. (**H**) Western blot analysis of phosphorylated claudin-5 after end of TTFields treatment. (**I**) Quantification of normalized relative levels of phosphorylated claudin-5 compared with total claudin-5 levels from two to four Western blots for each condition. OD, optical density. The stated *p*-value refers to differences between TTFields 72 h and the corresponding 72 h time point after TTFields cessation.

To determine if TTFields-induced delocalization of claudin-5 is mediated by the GEF-H1/Rho/ROCK pathway, we evaluated changes in the phosphorylation of GEF-H1 on serine 885 (S885) and claudin-5 on T207 in cerebEND cells exposed to TTFields via Western blot analysis. Our data demonstrate that similar to their effect in cancer cells, TTFields application in cerebEND cells promoted a time-dependent increase in GEF-H1 S885 phosphorylation with peak levels appearing at 10 min and remaining stable through the examined treatment duration (Figure 2A,B). RhoA activation was assessed following TTFields application for 15 min. Its activity was significantly increased by TTFields (Figure 2C). Changes in claudin-5 phosphorylation paralleled GEF-H1 activation (Figure 2D). Subsequent evaluation of the activation of ROCK, which mediates the downstream effects of GEF-H1 on claudin-5, demonstrated significantly increased activity following TTFields application in cerebEND cells (Figure 2E). Collectively, these data show that ROCK is an essential component of signal transduction pathways linking TTFields-induced microtubule disruption to the delocalization of claudin-5.

To further visualize the involvement of ROCK in claudin-5 delocalization upon TTFields administration, cerebEND cells were treated with fasudil, a clinical agent known to inhibit ROCK [39], prior to immunofluorescence staining. With the application of 10 µM fasudil, the distribution of claudin-5 in the cells to which TTFields were applied appeared more like untreated control cells that did not receive any treatment, than to cells that were subjected to TTFields but not fasudil (Figure 2F).

### 3.4. TTFields Effects on the CerebEND Morphology Were Reversible following a Recovery Period

We proceeded to inquire if the effects of TTFields were reversible. Following treatment, cells were allowed to recover at 37 °C for 24, 48, 72, and 96 h. Indeed, fluorescence microscopy images of cells stained with antibody against claudin-5 revealed that cell structures had already begun to normalize after 48 h. The narrow, elongated shapes of the cells, which were disrupted by TTFields, started to reappear. Maximum recovery of the cell structure was observed after 96 h (Figure 2G). We also examined the phosphorylation levels of claudin-5 immediately at treatment end and following 24–72 h recovery periods. TTFields treatment significantly increased claudin-5 phosphorylation, which was completely reversed after 72 h (Figure 2H,I). In addition, to account for the effects of TTFields on cell proliferation, cells were counted during and post-TTFields treatment. No significant change in cell count was observed in treated cells compared to the control (Figure S1A). Moreover, considering that apoptosis plays an important role in tissue development and homeostasis, cells were subjected to TUNEL assay after TTFields treatment to determine whether apoptosis was induced in the noncancerous cerebEND cells. Staurosporine-induced apoptotic cerebEND cells served as the positive control. No apoptosis was observed among untreated controls or cells treated with TTFields (Figure S1B).

### 3.5. TTFields at 100 kHz Altered BBB Integrity and Permeability and Switching from 100 to 200 kHz TTFields Frequency Maintained the Opened Barrier

**Figure 3:**
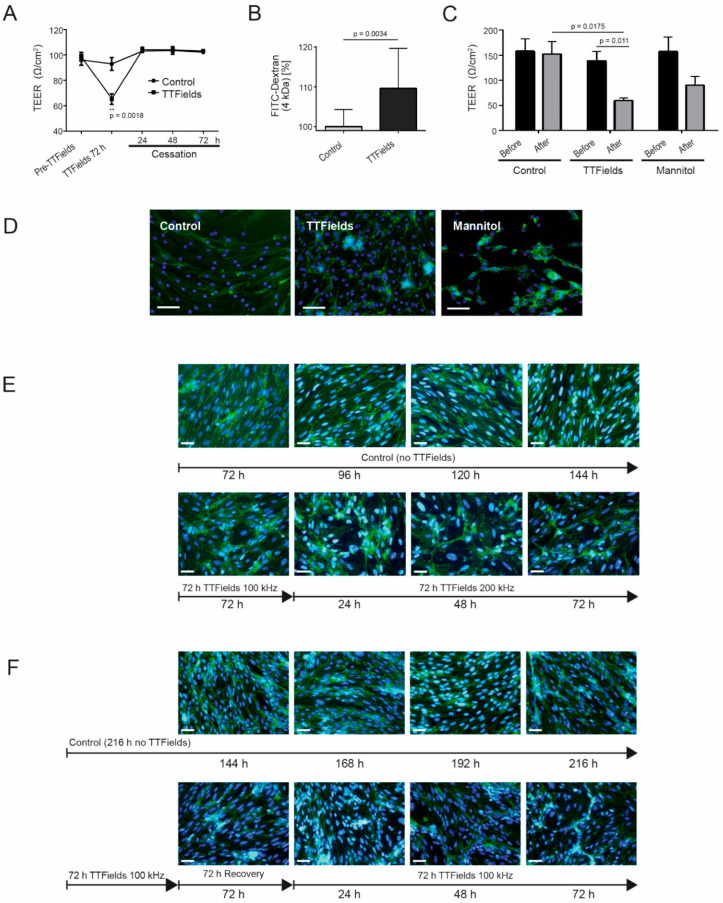
While TTFields did not affect cerebEND cell viability, they altered BBB integrity and permeability repeatedly. (**A**) TEER was measured before (pre-TTFields), directly after 72 h treatment with TTFields at 100 kHz, and during a 72 h recovery period (cessation). Asterisks indicate significant differences compared to the control of the same time point. (**B**) TTFields effects at 100 kHz on cell permeability were evaluated by a FITC-dextran permeability assay. (**C**) cerebEND cells were treated with either TTFields at 100 kHz for 72 h or 1.4 M mannitol for 24 h. The values shown in (**A**–**C**) are the mean of three independent experiments. Statistical significance was evaluated using an unpaired two-tailed Student’s *t*-test. (**D**) Immunofluorescence staining of cells under the same treatment conditions. (**E**) cerebEND cells treated with TTFields at 100 kHz for 72 h followed by 200 kHz for 24–72 h. Images shown are post-100 kHz TTFields treatment. (**F**) cerebEND cells treated with TTFields at 100 kHz for 72 h followed by recovery for 72 h and a repeated 100 kHz treatment for 72 h. Images shown are post-72 h recovery from the first and succeeding second treatment from 24 to 72 h. Cells shown in (**D**–**F**) were stained with claudin-5 antibody and conjugated to Alexa Fluor 488 (green). Nuclei were stained with DAPI (blue), magnification 40×, scale bars = 50 (**D**) and 200 µm (**E**,**F**), respectively. Shown are representative images of *n* = 3 independent experiments.

Integrity and permeability are two important determinants of an intact or compromised barrier. Transendothelial electrical resistance (TEER) is a very sensitive and reliable method to confirm the integrity and permeability of cell monolayers and the method of choice for assessing cell barrier integrity prior to drug transport evaluation [40]. Hence, we asked how TEER was affected by TTFields. After treating cerebEND cells with TTFields at 100 kHz for 72 h, TEER was measured and found to be significantly reduced relative to the control (*p* = 0.0018) (Figure 3A). TEER values rapidly reverted back to control levels, once TTFields were switched off (Figure 3A). In addition, following TTFields application, there was a significant increase (*p* = 0.0034) in the ability of 4 kDa FITC-dextran to permeate through the barrier relative to the control in this setting (Figure 3B). To further appraise the action of TTFields on BBB permeability, we compared their effects with hyperosmotic mannitol. The TEER measurements as well as immunofluorescent staining of the tight junction protein claudin-5 showed that the effects of TTFields on BBB integrity were comparable to mannitol (Figure 3C,D). However, the effects of TTFields were statistically significant, whereas mannitol’s effects were not (Figure 3C). Nonetheless, more drastic disruption of the cellular morphology could be observed in cerebEND cells treated with mannitol compared to TTFields (Figure 3D).

Since the TTFields frequency approved to therapeutically treat GBM patients is 200 kHz [28], we proceeded to examine the outcome of switching from one TTFields frequency to another. Moreover, we investigated the possibility of consecutive TTFields application subsequent to a period of recovery. Following treatment of cells with TTFields at 100 kHz for 72 h, the frequency was raised to 200 kHz, and the effect on cells was examined every 24 h for a total of 72 h. Claudin-5 immunofluorescence staining demonstrated that the barrier remained open after transitioning TTFields frequency from 100 to 200 kHz. Interestingly, the cells appeared similar in morphology after TTFields treatment at 100 kHz for 72 h and succeeding treatment at 200 kHz from 24 to 72 h (Figure 3E).

Next, a one-time repetition of TTFields administration was carried out. After subjecting cerebEND cells to TTFields at 100 kHz for 72 h, the cells were allowed to recover for 72 h before TTFields application with the same conditions was repeated. The similarity in cell morphology observed between the first TTFields application (Figure 1D) and the second, as visualized by claudin-5 staining, revealed that opening of the barrier through TTFields application could be repeated without impacting their effects (Figure 3F).

Altogether, our in vitro data suggest that TTFields open the BBB, indicating a possible clinical translation. Thus, we asked if we could confirm our in vitro findings in vivo.

### 3.6. TTFields Increased Vessel Permeability and Disrupted Microvessel Structure in Rat Brain

**Figure 4:**
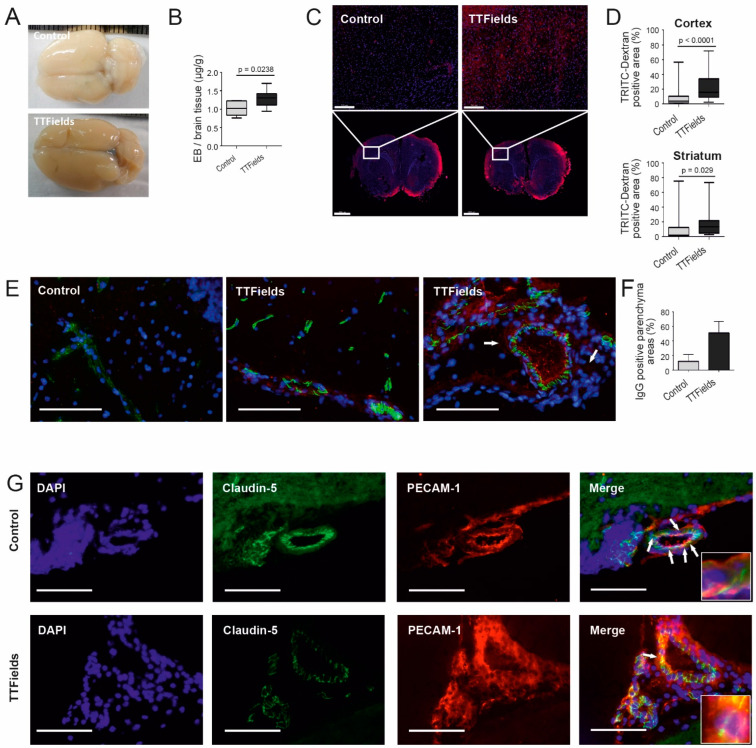
TTFields at 100 kHz for 72 h increased vessel permeability. (**A**) Whole brain images from rats injected intravenously with EB following 72 h treatment with TTFields (*n* = 10) or sham heat (*n* = 6). (**B**) Quantification of EB extracted from perfused rat brain following treatment based on absorbance measurements at 610 nm. (**C**) TD (red) accumulation in the brain parenchyma following TTFields. Nuclei were stained with DAPI (blue). Scale bars = 200 (upper) and 2000 µm (lower), respectively. (**D**) Uptake of TD by the cortex (upper) and the striatum (lower) of treated animals, as evaluated by measuring the TD autofluorescence. (**E**–**G**) Immunofluorescence staining of rat brain cryosections. (**E**) Cells were stained with antibody against TJ protein claudin-5 conjugated to Alexa Fluor 488 (green) and IgG (red). The control and the first TTFields image show longitudinal sections of blood vessels. The right image shows a cross-section of a TTFields-treated vessel. Arrows indicate delocalization of TJ proteins and areas of IgG extravasation. (**F**) Quantification of IgG extravasation into the brain parenchyma. Slide preparations were photographed under the fluorescence microscope and the percentage of IgG-positive parenchyma areas was calculated. (**G**) Double staining of claudin-5 (green) with PECAM-1 (red), producing a merged image of yellow within points of colocalization, which are indicated by arrows and shown in high magnification in the inlays. Nuclear staining with DAPI (blue). Magnification 40×, scale bar = 100 µm.

In order to evaluate BBB permeability in animals, healthy rat brains were assayed with Evans Blue (EB, 65 kDa) and TRITC-dextran (TD, 4 kDa). After treatment with TTFields at 100 kHz for 72 h, increased vessel permeability of EB into the rat brain could be observed through the distinct appearance of blue color (Figure 4A). Quantification of EB extracted from the brains revealed a significant difference between the control and treatment (*p* = 0.02) (Figure 4B). Similarly, increased vessel permeability of TD into the rat brain after TTFields treatment at 100 kHz for 72 h could be detected based on immunofluorescence images. A marked increase in TD accumulation was notable in TTFields-treated brains compared to control, both in the cortex and striatum (Figure 4C,D).

Additionally, rat brain cryosections were stained for the TJ protein claudin-5 and the intercellular junction protein PECAM-1 to visualize microvessels as well as double stained with immunoglobulin G (IgG) to examine BBB leakiness. Immunofluorescence of claudin-5 staining exhibited disrupted microvessel structure and IgG permeation after treatment with TTFields at 100 kHz for 72 h (Figure 4E). Claudin-5, which normally outlines microvessel walls, was delocalized. The microvessels appeared to have breaks or openings within their walls. Quantification of immunofluorescence photographs revealed increased IgG leakage by 35% in the samples treated with TTFields relative to the control (Figure 4F). Upon examination of split-channel images of the rat cryosections, PECAM-1 staining was more defined along the vessel lumen and the microvessel boundaries in the samples treated with TTFields compared to untreated control (Figure 4G). Moreover, in the samples to which TTFields were applied, the staining appeared dispersed. This could be evidence of reduced colocalized points of contact between claudin-5 and PECAM-1 (Figure 4G).

### 3.7. TTFields Increased Gadolinium Accumulation in the Rat Brain

**Figure 5:**
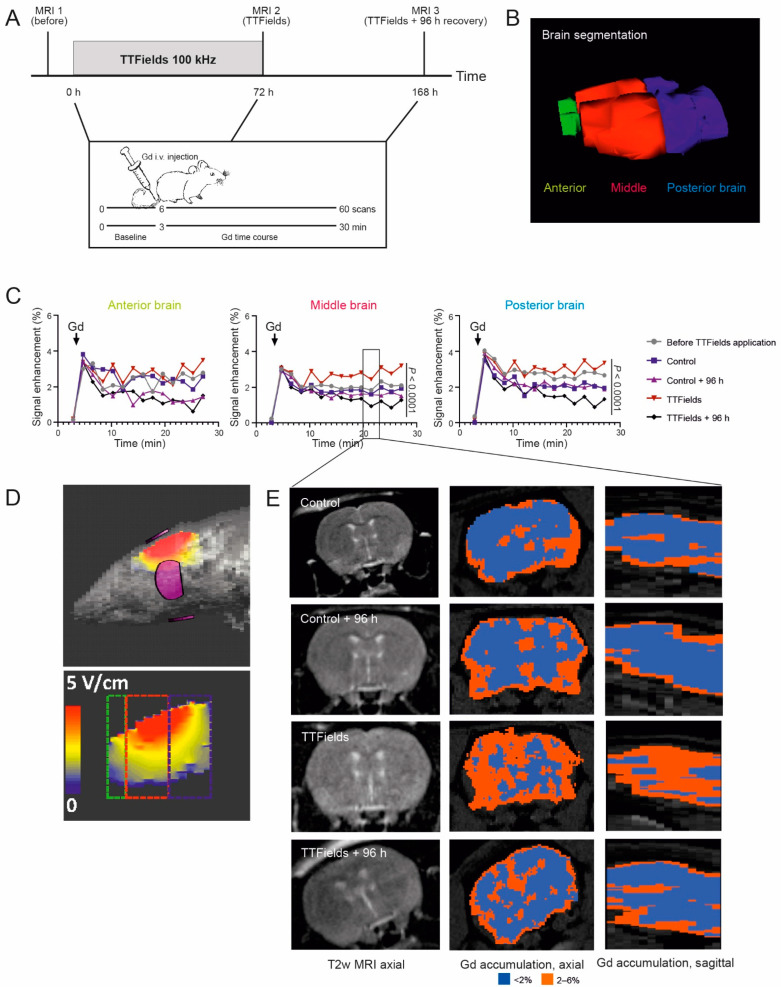
TTFields treatment led to transient Gd uptake into the rat brain. (**A**) Schematic representation of the experimental setup. (**B**) 3D representation of the brain segmentation: green = anterior, red = middle, and blue = posterior brain. (**C**) Time course DCE-MRI of Gd contrast agent in the anterior, middle, and posterior segmented areas of the brain before and after treatment with TTFields at 100 kHz for 72 h as well as 96 h after the end of treatment. In the middle and posterior parts of the brain, there was a significant Gd accumulation in the rats treated with TTFields approximately 10 min post-injection (*n* = 7) compared with the control animals (*n* = 3) (paired *t*-test, *p* < 0.0001). (**D**) Location of the two pairs of electrodes relative to the rat brain (upper) and simulations of TTFields delivery at 100 kHz, where the electrical field intensities’ distribution within the brain is shown (lower). The colored boxes indicate the areas defined as anterior (green), middle (red), and posterior (blue) brain. (**E**) T2-weighted MRIs (left) and Gd distribution 20–23 min post-Gd injection in control and rat brains treated with TTFields at 100 kHz in axial (middle) and sagittal view (right). Blue areas represent <2% and red areas 2–6% Gd enhancement.

To further investigate the permeation of molecules from the blood into the brain via treatment with TTFields, serial DCE MRI was performed. The same rats were analyzed before TTFields application after 72 h of TTFields treatment at 100 kHz and 96 h after treatment cessation (Figure 5A). Based on the MRI images, brains were digitally segmented into anterior, middle, and posterior parts (Figure 5B). In rats post-TTFields, the results demonstrated significantly increased signal enhancement in the middle and posterior brains approximately 10 min after intravenous Gd contrast agent injection, implying increased Gd accumulation (*p* < 0.0001) (Figure 5C). On the other hand, no significant Gd accumulation was observed in the anterior part of the rat brain. The differences in the Gd accumulation in the three parts of the rat brain were in accordance with the predicted electrical field intensities in each region as simulated using a rat model. Model calculations attributed a field of 2.7 ± 1.7 and 2.1 ± 1.2 V/cm RMS for the middle and posterior brains and 1.5 ± 0.6 V/cm RMS for the anterior brain (Table 1, Figure 5D). These simulated electrical fields were consistent with the electrical field measured in a rat brain using a probe (Table 1). No significant Gd accumulation was observed in the control groups of sham heat, animals injected with Gd pre-TTFields, or after 96 h of treatment cessation. The absence of Gd accumulation in brains after cessation points to the recovery of the BBB as shown in vitro. Analysis of the spatial distribution of Gd accumulation 20–23 min postcontrast administration showed that the Gd enhancement was distributed in the whole brain (Figure 5E), whereas control brains and brains after 96 h recovery showed minimal signal enhancement.

### 3.8. Combination of TTFields and Paclitaxel Decreased Tumor Volume and Tumor Cell Proliferation in Rats

**Figure 6:**
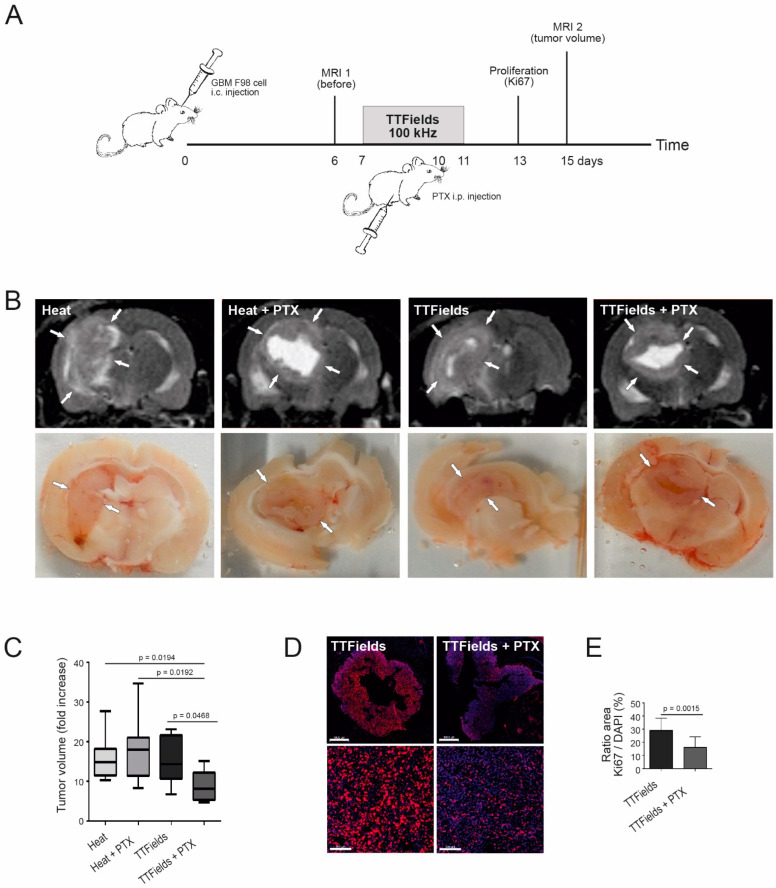
Combined TTFields and PTX treatment of rats. (**A**) Schematic representation of the time course. Rats (seven to eight animals per group) were injected orthotopically with GBM F98 cells. PTX (15 mg/kg) or vehicle were injected intraperitoneally on day 3 of TTFields at 100 kHz. (**B**) MRI scans (upper) and postmortem tissue sections (lower) at day 15 after tumor injection. Arrows indicate the tumor boundaries. (**C**) Quantification of the fold tumor volume increase in the different treatment groups. (**D**) Ki67 (red) and DAPI (blue) staining of combined TTFields-PTX treatment in the GBM tumor 3 days post-intraperitoneally 25 mg/kg PTX administration (3–4 rats per group). Scale bars = 1000 (upper) and 100 µm (lower), respectively. (**E**) Quantification of the Ki67/DAPI ratio showing decreased cell proliferation in the GBM tumor with the combined treatment.

PTX, a drug which has been used to treat malignant glioma and brain metastases [41,42], is normally not able to pass the BBB [43] and is quickly effluxed by the P-glycoprotein transporter; therefore, it is not effective in patients [44,45,46]. In our aim to further assess the ability of TTFields to transiently open the BBB, we treated tumor-induced rats with TTFields followed by PTX (Figure 6A). The fold tumor volume increase in rats treated with TTFields followed by PTX was significantly reduced (*p* < 0.05) compared to rats treated with TTFields alone, control sham heat alone, as well as heat and PTX combined (Figure 6B,C). These data point to an opening of the BBB by TTFields that allowed PTX to act on the tumor cells. In addition, this observation was supported by the cell proliferation marker Ki67/DAPI ratio, which was significantly reduced (*p* = 0.0015) in TTFields- and PTX-treated rats compared to TTFields alone, alongside a strongly reduced proliferation rate of tumor cells after the combined treatment (Figure 6D,E).

## 4. Discussion

The restrictiveness of the BBB remains the greatest challenge in delivering drugs to the CNS, as it impedes the passage of most chemotherapeutic agents, which have molecular masses ranging from 200 to 1200 Da. Although various techniques have been developed to overcome the BBB and enable drug delivery, such as physical modalities (e.g., FUS and PEFs) [47,48,49], enhanced lipid- and water-solubility through drug design, as well as employing carriers and vectors [50,51,52], these strategies still fall short in outcomes. For instance, it has been reported that issues with reproducibility of FUS procedures can be an obstacle to its clinical use [53]. Mannitol, a cell-impermeable nontoxic alcohol, has been used successfully for reversible opening of the BBB in hyperosmotic concentrations, both experimentally and clinically [50,51,52]. However, side effects, such as seizures and headaches, were noted to occur [54]. Most of these strategies come with drawbacks and risk factors for patients such as nontargeted delivery [54,55,56,57,58]. Accordingly, alternative strategies for transient opening of the BBB to enable delivery of drugs are needed to better treat patients. Relative to previously explored strategies, the favorable safety profile of TTFields may increase the likelihood of clinical adoption as a method for improved drug delivery.

To our knowledge, we are the first to report the effects of TTFields in a BBB model system composed of nonmalignant, healthy cells. Although 200 kHz is the optimal frequency to treat glioma cells and 150 kHz to treat breast, pancreatic, and nonsmall cell lung cancer [21,22,23], our study illustrates that application of TTFields at 100 kHz led to the transient and reversible opening of the BBB in vitro and in vivo. This finding paves the way for a future strategy whereby a combination of two TTFields frequencies (i.e., 100 and 200 kHz) will be employed for GBM treatment. The lower 100 kHz frequency could be used to open the BBB for enhanced drug delivery, followed by a switch to 200 kHz to target the proliferating GBM cells.

As with the cytotoxic effects of TTFields, our in vitro data revealed a dependence on treatment duration and electric field intensity. TTFields application at a frequency of 100 kHz for at least 72 h induced optimal effects on cerebEND cells, although some morphological changes were observed starting at 24 h. In addition, the cellular morphological distortions identified using claudin-5 immunofluorescence staining of cerebEND treated with TTFields of varying intensities leads us to hypothesize that a localized opening of the BBB for therapeutic intervention could be intensity dependent. It was established that there was a significant dose–response relationship concerning TTFields and tumor growth reduction [23,27]. With this in mind, targeted or localized opening of the BBB through TTFields for concomitant therapeutic strategies could be made possible through directed appropriation of suitable field intensities.

The mechanism through which TTFields mediate their effects involves the delocalization of the TJ proteins ZO-1 and claudin-5, the latter being the major TJ protein expressed by cerebEND cells [37], but not PECAM-1, disrupting the seal between the endothelial cells and forming gaps permeabilizing the BBB. As our results demonstrated, claudin-5 expression is unaltered by TTFields, thus leading to the assumption that claudin-5 delocalization accounts for the observed increased BBB permeability. While claudin-5 can be found both in tight BBB capillaries as well as in leaky capillaries with discontinuous junctions, it has been suggested that its expression is associated with endothelial lineage rather than leaky capillaries [59]. Altogether, this supports our findings that the application of TTFields modifies the assemblage of TJ proteins forming the barrier but not their expression levels.

The TJ complexes disintegrate due to the redistribution of claudin-5, and this leads to loss of the junctional barrier function, which allows the BBB to open. It appears that the cell monolayer remains intact, while the cell-to-cell connectivity is disturbed most probably due to the delocalization of TJ proteins normally coupled to the actin cytoskeleton. For this reason, we hypothesized that TTFields exert their effects by altering the cytoskeleton architecture, as was recently demonstrated [30]. Consistent with the aforementioned hypothesis, our results suggest that TTFields-induced ROCK activation can mediate phosphorylation of claudin-5 at T207. This phosphorylation event plays a key role in the transient nature of TTFields-induced BBB opening. Our findings exhibited reversion of endothelial cells to their original morphology starting 48 h post-TTFields cessation and completing after 96 h, with a concomitant reduction in phosphorylated claudin-5.

The relevance of this pathway was corroborated with immunofluorescence staining of cerebEND cells, whereby inhibition of ROCK with fasudil reversed the effects of TTFields on claudin-5. These findings are similar to a study in polarized intestinal epithelial cells, where inhibition of ROCK by Y-27632 led to increased paracellular permeability but did not show any change in distribution of intercellular junction proteins [60].

**Figure 7:**
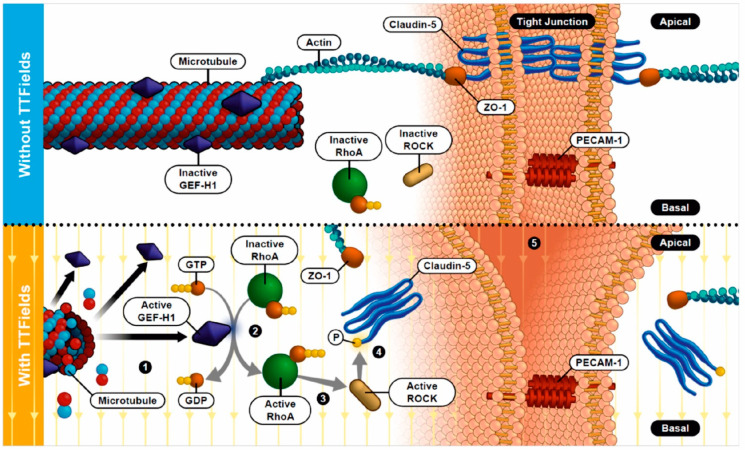
Scheme of the putative TTFields’ mode of action for opening the BBB. Without TTFields, claudin-5 forms tight junctions between the brain vascular endothelial cells and is connected via ZO-1 to the actin cytoskeleton (upper). With the use of TTFields, (1) microtubule organization is altered, activating GEF-H1. This leads to (2) increased levels of RhoA which, in turn, (3) activates ROCK resulting in (4) claudin-5 phosphorylation. Due to this, claudin-5 anchorage to ZO-1 is disrupted, causing claudin-5 to be delocalized from the cell membrane to the cytoplasm, thus (5) weakening the tight junctions (lower).

Comparing the mechanism of action of TTFields-induced permeability of the BBB with other modalities used to disrupt the BBB, a similarity in the processes targeting junction proteins at play can be observed. For instance, focused ultrasound induces the phosphorylation of Akt, whose activation alters cytoskeletal as well as tight junctional organization leading to BBB permeability [61]. Likewise, PEFs, specifically nanosecond PEFs, activate mitogen-activated protein kinases, which hyperphosphorylated the gap junction protein connexin 43 [62]. Osmotic opening of the BBB through infusion with a hypertonic solution, such as mannitol, is accomplished by dehydration of endothelial cells leading to vasodilation and cytoskeletal contraction due to the increased intracellular calcium. The contraction stretches the tight junctions, increasing BBB permeability [63]. With the use of TTFields, phosphorylation of claudin-5 due to the activation of ROCK possibly leads to an alteration of the claudin-5 anchorage to ZO-1, causing claudin-5 to be delocalized from the cell membrane to the cytoplasm (Figure 7).

Even though a number of available drugs are effective against GBM cells [41,42], most of them cannot cross the BBB [43]. As evidenced by our data, the concomitant therapy of TTFields and PTX proved to be more effective than each of the single therapies, suggesting that alterations in BBB permeability enabled PTX, a drug that does not normally pass the BBB, to penetrate the rat brain. TTFields therefore represent a possible clinical approach to enable drug delivery through the BBB for treatment of CNS disorders and should be further investigated in pre-clinical and clinical settings.

Overall, our hypothesis of using 100 kHz TTFields to open the barrier and transition to 200 kHz to enable targeting of GBM appears reasonable and feasible. Moreover, since TTFields administration followed by a period of recovery and subsequent repetition of administration did not show any drastic alterations in our model, this strategy remains viable even when multiple treatments are required. By and large, results from this study are an important initial step towards clinical translation, which could in turn help alleviate current barriers to CNS drug delivery and better facilitate the treatment of brain tumors.

Transient opening of the BBB by TTFields at 100 kHz could have clinical implications beyond the treatment of brain tumors and may be beneficial for other CNS diseases in which the BBB hampers the delivery of drugs. The multitude of CNS disorders including depression, epilepsy, multiple sclerosis, neurodegenerative diseases like Alzheimer’s, neuropathic pain, and schizophrenia calls for novel approaches that improve therapeutic delivery to the CNS, with minimal associated toxicity. Nonetheless, further assessment of the mechanisms by which TTFields function is a fundamental next step to possible clinical translation of our data.

## Figures and Tables

**Table 1:** Simulation and measurement of the electrical field intensity distribution of TTFields at 100 kHz within the rats’ brain.

	Electrical Field Intensity (V/cm RMS)	
	Simulated	Measured
Whole brain	2.3 ± 1.5	2.4 ± 0.04
Anterior brain	1.5 ± 0.6	n.d.
Middle brain	2.7 ± 1.7	n.d.
Posterior brain	2.1 ± 1.2	n.d.

Values are ± standard deviation. n.d. = not determined.

## Data Availability

The authors confirm that the data supporting the findings of this study are available within the article and its Supplementary Materials. Raw data are available from the corresponding author upon reasonable request.

## Supplementary Materials

The following supporting information can be downloaded at: https://www.mdpi.com/article/10.3390/biom12101348/s1, Supplementary Methods; Figure S1: TTFields at 100 kHz do not alter cerebEND cell numbers. References [36,37,52,64,65,66,67,68,69,70,71] are cited in the supplementary materials.
