# Supplementary material for: Tumor Treating Fields (TTFields) Reversibly Permeabilize the Blood–Brain Barrier In Vitro and In Vivo
Source: Biomolecules. 2022 Sep 22;12(10):1348. doi: 10.3390/biom12101348 (PMC9599321; doi:10.3390/biom12101348)
Supplement: Supplementary file 1 [file biomolecules-12-01348-s001.zip › Supplementary Figure S1_d1.0.pdf]

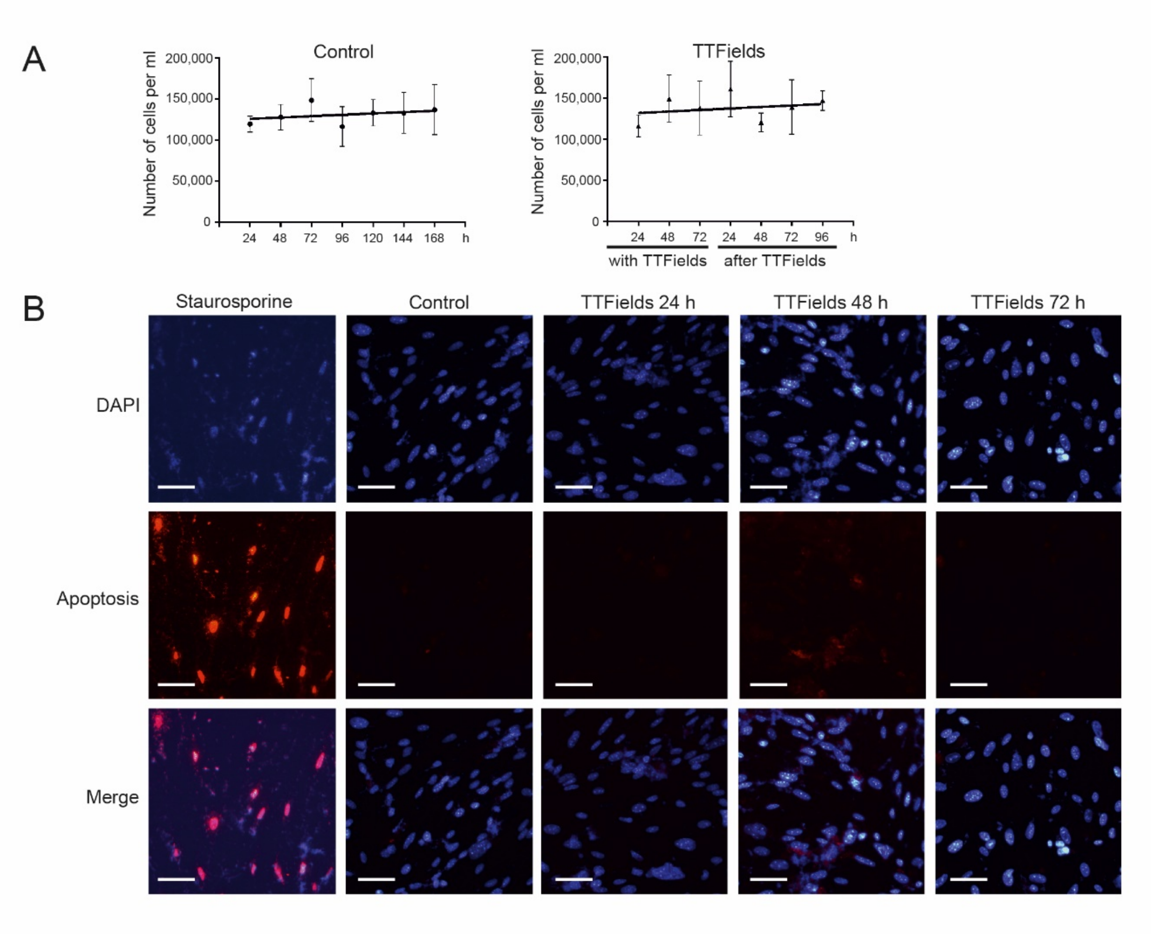

Figure S1. TTFields at 100 kHz do not alter cerebEND cell numbers. (A) Cell count of cerebEND cells during and after TTFields treatment. Values shown are the means of three independent experiments. (B) TUNEL assay of untreated control and TTFields-treated cerebEND cells. Apoptotic cells were stained red. Nuclear staining with DAPI (blue), magnification 40×, scale bar = 200 µm. Staurosporine-treated cells served as positive control. Images shown are representative of three independent experiments.
