# Supplementary material for: Tumor Treating Fields (TTFields) Reversibly Permeabilize the Blood–Brain Barrier In Vitro and In Vivo
Source: Biomolecules. 2022 Sep 22;12(10):1348. doi: 10.3390/biom12101348 (PMC9599321; doi:10.3390/biom12101348)
Supplement: Supplementary file 1 [file biomolecules-12-01348-s001.zip › Supplementary methods_23Aug2022_d1.0.pdf]

Supplementary Methods

Assessment of TTFields effects *in vitro*

Cell culture

Immortalized murine cerebellar microvascular endothelial cells (cerebEND) were generated in our lab [1,2] and cultured in Dulbecco's Modified Eagle's Medium (DMEM) (Sigma-Aldrich) supplemented with 50 U/ml penicillin/streptomycin and 10% fetal calf serum in a 37°C incubator (Forma™ Steri-Cult™ 200, Thermo Fisher Scientific, Inc.) until confluent. Cells were split in a 1:3 ratio once a week by dissociation with 0.25% Trypsin-EDTA (Gibco Thermo Fisher Scientific, Inc.) and medium was changed thrice per week. Cells were checked for contamination with mycoplasma monthly utilizing the Venor®GeM Classic Mycoplasma Detection Kit for conventional PCR (Minerva BioLabs).

TTFields application to cell cultures

Cells with a density of $4{\times}10^4$ were seeded onto 20 mm diameter cover slips (A. Hartenstein) laid in 6-well plates (Greiner Bio-One) for the control group or ceramic dishes (Novocure®) for the treatment group then grown in a 37°C incubator (Forma™ Steri-Cult™ 200) until confluent. Next, cells of the treatment group were subjected to TTFields application (100–300 kHz frequency) using the inovitro™ TTFields Lab Bench System (Novocure®) for 24–72 h, as described previously [3]. Following treatment, cells were allowed to recover in the 37°C incubator for 24–96 h.

To assess the effects of a transition from 100 to 200 kHz TTFields frequency, cerebEND cells were subjected to TTFields at 100 kHz for 72 h, which was then switched to 200 kHz for another 24–72 h. To assess whether TTFields application could repeatedly open the BBB, cerebEND cells treated with TTFields for 72 h were allowed to recover for 72 h and then treated again for 72 h.

In addition, to test the effects of varying TTFields intensities, cerebEND cells were subjected to TTFields at 100 kHz with intensities of 1.62, 0.97, and 0.76 V/cm RMS for 24–72 h, respectively.

Cell counting

After treatment, cells were washed with phosphate buffered saline (PBS) (Sigma-Aldrich®) and dissociated with 0.25% Trypsin-EDTA (Gibco Thermo Fisher Scientific Inc.). Upon trypsinization, cells were vortexed and loaded onto the Scepter 2.0 Cell Counter device (Merck Life Science, Merck KGaA) for counting.

Immunofluorescence staining of cerebEND

Cells were seeded onto 20 mm cover slips and grown until confluent. Next, they were washed 3× with PBS and fixed with ice-cold methanol for 20 min at –20°C. Cells were again washed 3× with PBS and blocked in PBS containing 5% donkey serum (DS, Abcam, plc.) for 1 h at room temperature. Afterwards, cells were incubated with the primary antibodies mouse anti-claudin-5 conjugated to Alexa Fluor 488 (1:500, Thermo Scientific, Cat. No. 352588), mouse anti-zonula occludens-1 conjugated to Alexa Fluor 488 (1:500, Thermo Scientific, Cat. No. 339188) and rabbit anti-PECAM-1 (1:500, Novus Biologicals, Cat. No. NB100-2284) (in 1% bovine serum albumin [BSA]/PBS with 5% DS) overnight at 4°C. Then, the cells were washed 3× before probing with the secondary antibody anti-rabbit Alexa Fluor 555 (1:400 in 1% BSA/PBS with 5% DS, Invitrogen, Cat. No. A-21429) for 1 h at room temperature, if not already conjugated. Finally, they were mounted on slides using Fluoroshield Mounting Medium with DAPI (Abcam, plc.) and allowed to dry for microscopy.

Claudin-5 expression analysis

Expression of the TJ protein claudin-5 was assessed using Western blot. Briefly, cells were washed 2× with PBS and lysed with RIPA buffer (50 mM Tris pH 8.0, 150 mM NaCl, 0.1% SDS, 0.5% sodium deoxycholate, 1% NP40) containing protease inhibitor cOmplete Ultra Tablets Mini (Roche) and Phenylmethylsulfonylfluoride (PMSF, Sigma-Aldrich). Cells were afterwards sonicated (SONOPULS; BANDELIN) and mixed with Laemmli buffer containing 5% β-mercaptoethanol (Sigma-Aldrich). After denaturation at 95°C, they were run through a 10% SDS-PAGE mini gel and blotted overnight using a Mini Trans-Blot Electrophoretic Transfer Cell (Bio-Rad). Subsequently, the membrane was blocked in 5% non-fat dry milk (Carl Roth) and probed with the primary antibodies for claudin-5 (1:500, Invitrogen, Cat. No. 35-2500) and phospho-claudin-5 (1:1,000, Thermofisher, Cat. No. PA5-105058) followed by secondary anti-mouse antibody (1:3,000, Roche Lumi Light Plus). Horse radish peroxidase-conjugated β-actin (1:2,500, Sigma-Aldrich, Cat. No. A3854) served as endogenous control.

Detection was carried out using an enhanced chemiluminescence solution [4] and viewed with ImagenFlourChem FC2 (Cell Biosciences, Inc.) with the AlphaView Software (Version 1.3.0.7, Alpha Innotech Corp.). Densitometric analysis was carried out using ImageJ (NIH). 10 µl of PageRuler Plus Prestained Protein Ladder (Thermo Fisher Scientific) was loaded as marker.

Cellular fractionation

Whole cell lysates were fractionated using the Sub-cellular Protein Fractionation Kit (Thermo Fisher) following manufacturer's instructions. Briefly, cells were dissociated using 0.25% Trypsin-EDTA (Gibco Thermo Fisher Scientific Inc.) and pooled in 2 ml microcentrifuge tubes (Eppendorf). Next, they were washed with PBS and centrifuged at 500 ×g for 3 min to obtain the cell pellet. Reagents were added followed by incubation and centrifugation steps to acquire the cytoplasmic and membrane fractions.

Transendothelial electric resistance measurement

Cells were grown on 24 well PET transwell-inserts (Corning, Inc.) with a pore diameter of 0.4 µm. Following TTFields treatment, transendothelial electric resistance (TEER) was measured with the volt-ohm meter device EVOM (World Precision Instruments). Blank filters were used as internal control.

Fluorescein isothiocyanate-dextran permeability assay

Upon reaching confluence, cells grown on transwell-inserts placed into high ceramic dishes (inovitro™ System; Novocure®) were subjected to TTFields. Afterwards, the inserts were transferred on a plate with each well containing 500 µl DMEM medium without phenol red (Thermo Scientific/Gibco) supplied with 10 ml L-glutamine (Thermo Scientific/Gibco) and washed with PBS. Next, 200 µl 4 kDa fluorescein isothiocyanate-dextran (FITC-dextran) (Sigma Aldrich) dissolved in the same medium was added to the insert. 100 µl of medium was taken out from each well after 1 h for measurement of fluorescence intensity at a wavelength of 485 nm and 535 nm excitation and emission, respectively, using a Tecan GENios Microplate Reader (MTX Lab Systems).

TdT-mediated dUTP-biotin nick end labelling assay

Cells were seeded onto cover slips and treated with TTFields. Afterwards, the occurrence of apoptosis within the cells was detected and quantified using the In Situ Cell Death Detection Kit (Roche) by following manufacturer's instructions. Briefly, cells were washed with PBS and fixed with 4% paraformaldehyde in PBS. Next, cells were permeabilized in 0.1% Triton X-100 and 0.1% sodium citrate solution, incubated in TdT-mediated dUTP-biotin nick end labelling (TUNEL) reaction mixture for 1 h at 37°C and washed with PBS. Finally, samples were analyzed by fluorescence microscopy. To induce apoptosis and serve as positive control, 5.3 mM Staurosporine (Sigma-Aldrich) was applied to cells and incubated for 24 h.

GEF-H1 activation

Cells were treated with TTFields at 100 kHz for 10-, 15-, 30- and 60-min. Cells were lysed, and a Western blot was performed and analyzed as described in the previous section. The following antibodies, diluted in 5% BSA, were used: GEF-H1 rabbit monoclonal antibody (1:1,000, Cell Signalling, Cat. No. 4145), phospho-GEF-H1 (Ser885) (1:1,000, Cell Signalling, Cat. No. 14143), and Glyceraldehyde 3-phosphate dehydrogenase (GAPDH) (1:500, Santacruz, Cat. No. sc-137179), followed by horseradish peroxidase-conjugated secondary antibody (1:2,000, Cell Signalling) and a chemiluminescent substrate (Sigma-Aldrich).

RhoA activation assay

To measure RhoA activity (GTP-bound active form), cerebEND cells were treated with TTFields at 100 kHz for 15 min. Cells were then lysed and RhoA activity was measured by the RhoA G-LISA activation assay in accordance with the manufacturer's instructions (Cytoskeleton). Results were expressed as the ratio of OD of treated cells to the OD of untreated cells normalized to total RhoA.

ROCK activation assay

To measure ROCK activity, cerebEND cells were treated with TTFields for 10-, 15-, 30-, and 60 min. Cells were then lysed, and ROCK activity was measured, utilizing the ROCK activity assay kit in accordance with the manufacturer's instructions (Abcam, plc.).

Treatment of cerebEND with Rho kinase inhibitor

cerebEND cells were seeded onto glass cover slips as aforementioned. Upon reaching confluence, 10 µmol/l fasudil [5] was added to the cells prior to TTFields administration at 100 kHz for 72 h for simultaneous treatment. Cells were stained with claudin-5 as described in the previous section.

Mannitol treatment

To further assess the effects of TTFields, we used hyperosmotic mannitol for comparison. Cells were treated with 1.4 M [6] mannitol (Covetrus) diluted in cell medium for 24 h. Afterwards, TEER was measured, and claudin-5 expression was assessed through immunofluorescence staining, as described in the previous sections.

Assessment of TTFields effects *in vivo*

Animals

The study was conducted using 10- to 14-week-old female Sprague Dawley (SD) rats of 200–250 g each (Envigo, Israel) or 12-week-old female Fisher rats of 160–180 g each (Envigo, USA) in compliance with the Israeli Animal Care and Use Committee (approval IL-19-6-266 and IL-19-8-335). No inclusion or exclusion criteria were applied.

Tumor Treating Fields

Healthy SD or Fisher rats bearing intracranial glioblastoma were treated with TTFields at 100 kHz as previously described [7]. Briefly, TTFields were delivered through two pairs of ceramic electrodes connected to a field generator to deliver alternating currents of 100 kHz. The electrodes are composed of high capacitance ceramic disks (lead magnesium niobate–lead titanate [PMN-PT]) of 13 mm by 9 mm in diameter. Each pair of electrodes was attached to the shaved rat head via conducting medical hydrogel. One pair was attached to the head and chin and one pair to the left and right cheek of the rat, generating two perpendicular alternating field directions. The current source output was switched every 1 s between the two pairs of electrodes. The temperature was constantly monitored by thermistor attached to each electrode. Control treatment was produced by sham heat electrodes that incorporate an electrical resistor producing equal temperature changes to those produced by the TTFields electrodes but do not deliver electrical fields.

Measurements and simulations of the electrical field intensities delivered by TTFields to the rat brain

Mathematical simulations of TTFields delivery and distribution to the rat brain were performed using Sim4life software (ZMT Zurich MedTech AG) and a rat brain model. Conductivity and permittivity of the various tissues were assigned according to the software database. To simulate TTFields delivery, a constant voltage was set between the arrays to generate current equivalent to those determined for the rats in this study. The field intensities were calculated for the anterior, middle, and posterior segments of the brains, as defined for the dynamic contrast-enhanced (DCE)-MRI.

To validate the simulations, field intensity measurements in the rat brain were carried out in the whole brain (Table 1). One set of electrodes was attached to the head and chin of a SD rat.

The electrical field intensities were measured parallel to plane between the electrodes, with a coaxial probe (2 mm in diameter and 2 mm distance between the probe tips) inserted vertically into the middle brain through a hole in the skull. Measurements of the electric field's intensities were done using a floating scope (Fluke, 199C).

Evans blue and TRITC-dextran permeability assays

Three days after treatment start, SD rats were intravenously injected with 2 mg/kg of 2% EB solution (Sigma-Aldrich). The EB was allowed to circulate for 2 h, then treatment was stopped, followed by intracardiac perfusion with cold saline. EB was extracted from the brains after tissue homogenization and the addition of trichloroacetic acid solution (Sigma-Aldrich) in a ratio of 1:3. Quantification was done at 610 nm with an Infinite F200 Tecan (Life Sciences) plate reader [8]. Likewise, at the end of TTFields, rats were intravenously injected with 100 mg 4 kDa TRITC-dextran (TD, TdB Labs, AB) in 1 ml saline. After 2 min, brains were harvested and properly oriented in a cryomold then freshly snap-frozen in O.C.T. compound (Tissue-Teck®) to avoid diffusion and wash-out of the dextran [9]. Brains were cryo-sectioned to 14 µm thickness. Sections were fixed in acetone and frozen until use.

For quantifying TD, sections were only stained with DAPI and then scanned at magnification 20× with a Pannoramic flash 250 digital scanner (3DHistech, Ltd.). Two regions of interest of 1.5 mm^2, one in the cortex area and one in the striatum area, were saved using the software CaseViewer version 2.2 (3DHistech, Ltd.), and the percentage of TRITC fluorescence among the total area was quantified using ImageJ 1.52p (NIH).

Immunofluorescence staining of rat brain

To visualize brain microvessel structures, cryosections were thawed for 20 min at room temperature after which they were fixed in ice-cold methanol for 20 min at 20°C. Samples were air dried for 10 min at room temperature and washed with PBS for 5 min each. Tissue permeabilization followed using 1 ml 0.5% Triton-X in PBS for 7 min. Slides were washed 3× with PBS for 5 min each. Next, the samples were air dried then blocked with 10% DS in PBS for 90 min. Antibody incubation was performed as described above for cerebEND cells. In addition, antibodies directed against IgG (rabbit; Abcam) were utilized in 1:500 dilution. The secondary antibody goat anti-rabbit Alexa Fluor 555 (Invitrogen, 1:400) was used as described above. Thereafter, slides were washed twice with PBS for 30 min each followed by distilled water for 20 min. Next, slides were air dried for 5 min at room temperature and mounted as

described. For quantification of IgG-positive parenchyma, a total of 295 images were visually scored for the presence of IgG outside the vessel lumen. Percentages were calculated for both control and treatment groups.

Brain sections of GBM-bearing rats were incubated for 1 h with primary antibody Ki67 (rabbit; Cell Marque, 1:200) at room temperature in a humidity chamber, washed, incubated with fluorescence Cy™3-AffiniPure donkey anti-rabbit IgG secondary antibody (H+L) (1:200, Jackson ImmunoResearch Laboratories, Inc.) for 1 h and DAPI (Biolegend) as a counterstain. At least two regions of interest on the tumor rim were processed. The Ki67 tumor cell proliferation ratio was quantified using ImageJ (NIH) and defined as the percentage of positive nuclear staining among the total nuclear surface.

Serial dynamic contrast-enhanced MRI

To further detect increase in permeability, serial DCE MRI were acquired at three time points for each SD rat: before treatment with TTFields, after 72 h of treatment, and 96 h post treatment. Rats were placed in a dedicated head coil and MRI-scanned using a 1 Tesla ICON MRI system (Bruker). For DCE-MRI, 60 T_1 weighted images were continuously acquired for a total time of 30 min, with acquisition time of 29 s for each image. The gadolinium-DTPA (Gd)-based MRI contrast agent Magnetol (Soreq Radiopharmaceuticals) was intravenously injected at a dose of 0.1 mmol/kg after the sixth image of each series. The MRI protocol used was a gradient echo sequence with time to repetition (TR) 180 ms, time to echo (TE) 2.7 ms, flip angle 60°, 14 transverse slices of 1.25 mm thickness, Field of view (FOV) 32 mm × 32 mm, matrix 128 × 128, in plane resolution of 250 µm × 250 µm. Previously to DCE-MRI, anatomical T_2 weighted images were acquired using a rapid acquisition with relaxation enhancement (RARE) sequence with TR 3050 ms, TE 85 ms and same geometry and resolution as in DCE-MRI. DCE-MRI data analysis was performed by applying image registration (maximization of mutual-information method) [10] to each series of images in order to correct for misalignment of the images due to brain involuntary movement over the 30 min. For each image of the series, the percentage of contrast enhancement was calculated voxel-wise by subtracting the signal of the baseline (the average of the first six images of the series) from the signal of the image at each time point, dividing by the baseline and multiplying with 100. The front, middle and posterior segments of the brains were manually segmented using ITK-Snap software version 3.6.0 [11].

Segmentation contained mainly the dorsal parts of the brains, avoiding the ventricles and the brain borders. The average of the percent signal change in each brain segment over time was analyzed for Gd accumulation. Moreover, in order to visualize Gd spatial distribution, parametric maps of the median of the time points between 20 and 23 min post-Gd injection were created. All image calculations were done using home-made scripts written in Python software version 3.6 (Python Software Foundation).

TTFields and paclitaxel treatment

Fisher rats were intracranially implanted with F98 GBM cells (ATCC). A mixture of 10,000 cells in 5 µl of growth factor reduced high concentration matrigel matrix (1:1) (Corning, Inc.) were injected 2.5 mm lateral to the midline to the right side, 1 mm anterior to the coronal fissure and 6 mm deep from the skin surface using a stereotaxic system. Six days after tumor injection, TTFields or sham heat electrodes were placed on the rat head and treatment was initiated (7–8 rats per experimental group). 72 h later, while animals were still treated for an additional 24 h, PTX (15 mg/kg, TEVA) or vehicle (1:1 mixture of Kolliphor® EL and ethanol [99.8%]; both from Sigma-Aldrich) was injected intraperitoneally. For assessing the tumor volume, MRI scans were performed 1 day prior to TTFields administration as well as 4 days after treatment-end using a T_2 weighted protocol (MRI parameters: RARE sequence with TR of 3,000 ms, TE of 95 ms, 12 transverse 1 mm thick slices, six averages, FOV 30 mm × 30 mm, in-plane resolution of 234 µm × 234 µm and acquisition time of 3 min). The tumor volume for each animal was obtained by manually compiling tumor areas from all slices that contained tumors using ITK-SNAP software.

In a second experiment, rats were treated as described above, but three days after PTX injection (intraperitoneally 20 mg/kg), the rat brains were freshly snap-frozen in over-the-counter compound (Tissue-Teck®) for assessing tumor cell proliferation with Ki67 marker by immunohistochemistry as described above.

References

1. Silwedel, C.; Forster, C. Differential susceptibility of cerebral and cerebellar murine brain microvascular endothelial cells to loss of barrier properties in response to inflammatory stimuli. *J Neuroimmunol* **2006**, *179*, 37-45, doi:10.1016/j.jneuroim.2006.06.019.

2. Burek, M.; Salvador, E.; Forster, C.Y. Generation of an immortalized murine brain microvascular endothelial cell line as an in vitro blood brain barrier model. *J Vis Exp* **2012**, e4022, doi:10.3791/4022.

3. Porat, Y.; Giladi, M.; Schneiderman, R.S.; Blat, R.; Shteingauz, A.; Zeevi, E.; Munster, M.; Voloshin, T.; Kaynan, N.; Tal, O.; et al. Determining the Optimal Inhibitory Frequency for Cancerous Cells Using Tumor Treating Fields (TTFields). *J Vis Exp* **2017**, doi:10.3791/55820.

4. Whitehead, T.P.; Kricka, L.J.; Carter, T.J.; Thorpe, G.H. Analytical luminescence: its potential in the clinical laboratory. *Clin Chem* **1979**, *25*, 1531-1546.

5. Yin, L.; Morishige, K.; Takahashi, T.; Hashimoto, K.; Ogata, S.; Tsutsumi, S.; Takata, K.; Ohta, T.; Kawagoe, J.; Takahashi, K.; et al. Fasudil inhibits vascular endothelial growth factor-induced angiogenesis in vitro and in vivo. *Mol Cancer Ther* **2007**, *6*, 1517-1525, doi:10.1158/1535-7163.MCT-06-0689.

6. Linville, R.M.; DeStefano, J.G.; Sklar, M.B.; Chu, C.; Walczak, P.; Searson, P.C. Modeling hyperosmotic blood-brain barrier opening within human tissue-engineered in vitro brain microvessels. *J Cereb Blood Flow Metab* **2020**, *40*, 1517-1532, doi:10.1177/0271678X19867980.

7. Shteingauz, A.; Porat, Y.; Voloshin, T.; Schneiderman, R.S.; Munster, M.; Zeevi, E.; Kaynan, N.; Gotlib, K.; Giladi, M.; Kirson, E.D.; et al. AMPK-dependent autophagy upregulation serves as a survival mechanism in response to Tumor Treating Fields (TTFields). *Cell Death Dis* **2018**, *9*, 1074, doi:10.1038/s41419-018-1085-9.

8. Wang, H.L.; Lai, T.W. Optimization of Evans blue quantitation in limited rat tissue samples. *Sci Rep* **2014**, *4*, 6588, doi:10.1038/srep06588.

9. Hoffmann, A.; Bredno, J.; Wendland, M.; Derugin, N.; Ohara, P.; Wintermark, M. High and Low Molecular Weight Fluorescein Isothiocyanate (FITC)-Dextrans to Assess Blood-Brain Barrier Disruption: Technical Considerations. *Transl Stroke Res* **2011**, *2*, 106-111, doi:10.1007/s12975-010-0049-x.

10. Wells, W.M., 3rd; Viola, P.; Atsumi, H.; Nakajima, S.; Kikinis, R. Multi-modal volume registration by maximization of mutual information. *Med Image Anal* **1996**, *1*, 35-51, doi:10.1016/s1361-8415(01)80004-9.

11. Yushkevich, P.A.; Piven, J.; Hazlett, H.C.; Smith, R.G.; Ho, S.; Gee, J.C.; Gerig, G. User-guided 3D active contour segmentation of anatomical structures: significantly improved efficiency and reliability. *Neuroimage* **2006**, *31*, 1116-1128, doi:10.1016/j.neuroimage.2006.01.015.

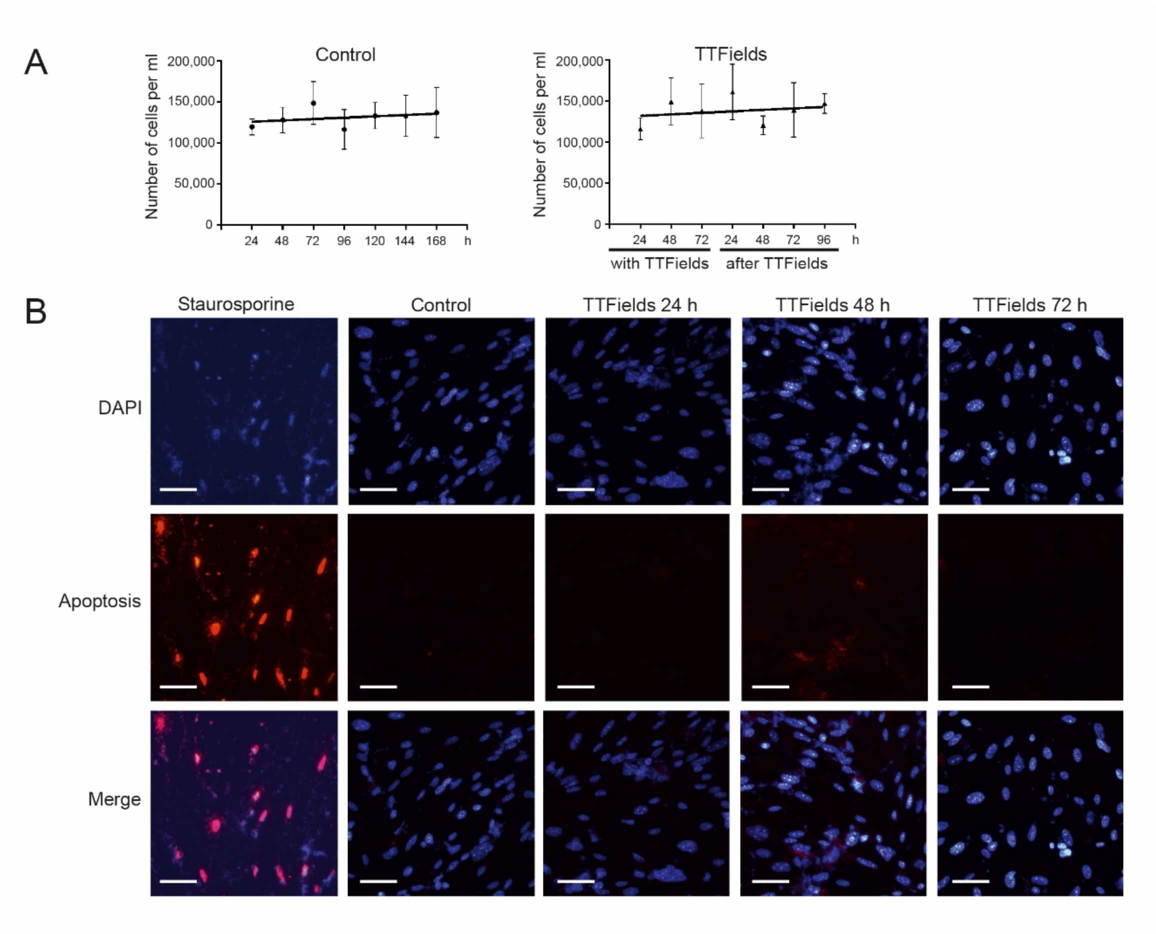

Figure S1. TTFields at 100 kHz do not alter cerebEND cell numbers. (A) Cell count of cerebEND cells during and after TTFields treatment. Values shown are the means of three independent experiments. (B) TUNEL assay of untreated control and TTFields-treated cerebEND cells. Apoptotic cells were stained red. Nuclear staining with DAPI (blue), magnification 40×, scale bar = 200 µm. Staurosporine-treated cells served as positive control. Images shown are representative of three independent experiments.
